# Space-based and object-centered gaze cuing of attention in right hemisphere-damaged patients

**DOI:** 10.3389/fpsyg.2015.01119

**Published:** 2015-08-04

**Authors:** Mario Dalmaso, Luigi Castelli, Konstantinos Priftis, Marta Buccheri, Daniela Primon, Silvia Tronco, Giovanni Galfano

**Affiliations:** ^1^Department of Developmental and Social Psychology, University of PadovaPadova, Italy; ^2^Center for Cognitive Neuroscience, University of PadovaPadova, Italy; ^3^Department of General Psychology, University of PadovaPadova, Italy; ^4^Human Inspired Technologies Research Center, University of PadovaPadova, Italy; ^5^Department of Rehabilitation, Unità Locale Socio Sanitaria 15, CittadellaItaly

**Keywords:** gaze cuing, object-centered attention, right hemisphere-damaged patients, hemispheric asymmetry, social cognition

## Abstract

Gaze cuing of attention is a well established phenomenon consisting of the tendency to shift attention to the location signaled by the averted gaze of other individuals. Evidence suggests that such phenomenon might follow intrinsic object-centered features of the head containing the gaze cue. In the present exploratory study, we aimed to investigate whether such object-centered component is present in neuropsychological patients with a lesion involving the right hemisphere, which is known to play a critical role both in orienting of attention and in face processing. To this purpose, we used a modified gaze-cuing paradigm in which a centrally placed head with averted gaze was presented either in the standard upright position or rotated 90° clockwise or anti-clockwise. Afterward, a to-be-detected target was presented either in the right or in the left hemifield. The results showed that gaze cuing of attention was present only when the target appeared in the left visual hemifield and was not modulated by head orientation. This suggests that gaze cuing of attention in right hemisphere-damaged patients can operate within different frames of reference.

## Introduction

The eyes of our conspecifics represent a privileged target for our attention, as shown by several recent studies (e.g., Birmingham et al., 2008; Levy et al., 2013; Boggia and Ristic, 2015). The prioritized processing of eye gaze stimuli might be related to the fact that they are a valuable source of information which provides important insights not only about where other individuals are attending to, but also about their internal states such as future intentions or beliefs (e.g., Baron-Cohen, 1995). This, in turn, can help us developing a better interaction with our social and physical environment (e.g., Emery, 2000; Shepherd, 2010).

The relevance of the eye gaze of others has been testified by a phenomenon known as gaze cuing of attention, which consists of the tendency to shift attention in the direction gazed by a face (for a review, see Frischen et al., 2007). This can be empirically investigated by asking participants to manually respond to a lateralized target that is preceded by the onset of a task-irrelevant centrally placed face with averted gaze. Shorter reaction times (RTs) are generally observed when the target appears at the same spatial location indicated by the gaze of the face stimulus, rather than when the target appears elsewhere (i.e., the gaze-cuing effect; see Friesen and Kingstone, 1998). This pattern of results confirms that individuals tend to shift attention towards the same direction indicated by eye gaze stimuli (see also Driver et al., 1999; Galfano et al., 2012). The intrinsic social nature of this type of behavior has recently been supported by several studies conducted both on healthy participants (e.g., Teufel et al., 2010; Schulz et al., 2014; Cole et al., 2015) and on clinical populations (e.g., Nestor et al., 2010; Dalmaso et al., 2013, 2015; Marotta et al., 2014). In particular, as for healthy participants, it has been shown that gaze cuing is strongly affected by social variables related to both the observer and the person observed, as well as to the relationship between the two individuals. For instance, group membership (e.g., Pavan et al., 2011; Ciardo et al., 2014; Chen and Zhao, 2015), social status/dominance (e.g., Jones et al., 2010; Dalmaso et al., 2012, 2014), political affiliation (Liuzza et al., 2011, 2013; see also Carraro et al., 2015), trustworthiness (e.g., Süßenbach and Schönbrodt, 2014), and participants’ age (e.g., Slessor et al., 2008; Kuhn et al., 2015), autistic traits (e.g., Senju et al., 2004; Bayliss et al., 2005; Ristic et al., 2005) or phobias (e.g., Pletti et al., 2015) can all impact gaze cuing of attention.

The great relevance of gaze in shaping human behavior prompted researchers to hypothesize the presence of a neurocognitive mechanism specifically devoted to gaze cuing of attention, although the results are not always consistent (e.g., Hietanen et al., 2006; Tipper et al., 2008; Nummenmaa and Calder, 2009). However, according to a recent neuroimaging study, the neural underpinnings of gaze cuing of attention seem to involve several brain areas related to gaze and face processing (Callejas et al., 2014). In more detail, these brain areas would first process sensory information conveyed by facial stimuli and, subsequently, this information would be passed to several regions involved in orienting of attention. Interestingly, these regions would mostly be located in the right hemisphere – which is well known to be specialized for face processing (e.g., Ojemann et al., 1992) – and would include the right-posterior superior temporal sulcus, the right-posterior intraparietal sulcus, and the right-inferior frontal junction.

The involvement of the right hemisphere in gaze cuing of attention has been also investigated in studies adopting a causal approach with both healthy individuals (e.g., Porciello et al., 2014) and neuropsychological patients. As for the studies with patients, Kingstone et al. (2000) observed gaze cuing of attention in two split-brain patients, but only when a lateralized eye gaze cue was projected towards the right hemisphere. Interestingly, when gaze cues were replaced with a non-social cue such as an arrow, the cuing effect was bilateral (Ristic et al., 2002). Other studies focused on patients with brain lesions which were specifically localized in the right hemisphere. In this regard, Akiyama et al. (2006) observed a preserved arrow cuing of attention in the face of an impaired gaze cuing of attention in a patient with a rare lesion circumscribed to the right superior temporal gyrus, which has been shown to be crucially involved in face and gaze processing (e.g., Allison et al., 2000). However, no clear conclusion about the eventual lateralization of the effects as a function of the visual hemifield can be drawn because only right-sided targets were tested because of the patient’s left hemianopia. Furthermore, Vuilleumier (2002) presented four right hemisphere-damaged patients with peripheral targets and eye gaze cues (Experiments 4 and 5) or arrow cues (Experiment 6). Even if left neglect was present in all participants, eye gaze stimuli elicited a reliable orienting detectable even in the contralesional side, whereas arrow cuing of attention was overall weaker. More recently, Bonato et al. (2009) tested right hemisphere-damaged patients (either with or without left neglect) by presenting centrally placed symbolic cues (i.e., arrows and numbers) and schematic eye gaze stimuli. Strikingly, in both groups (patients with or without left neglect) reliable orienting of spatial attention emerged in response to arrow cues but not to numbers, whereas eye gaze produced orienting of attention only in patients without left neglect.

All the aforementioned neuropsychological studies provided interesting insights regarding the functioning of a broad brain network that would support gaze cuing of attention. Even if a direct comparison of the findings of those studies is difficult because of the type of different brain lesions characterizing the patients and because of the adopted paradigms, a common feature of these studies is that they only focused on the space-based component of visual attention. Indeed, participants were presented with centrally placed faces (or eyes only) displayed upright and targets could generally appear either in the gazed-at location or in the opposite hemifield. This approach, however, does not allow to tease apart the contribution of two different modalities of attention shifting depending on specific reference frames. Indeed, on the one hand, attention mechanisms operate on simple spatial coordinates along hypothetic spatial vectors. However, we know that humans can shift their attention at least within another frame of reference, which is object-centered (e.g., Fink et al., 1997; Behrmann and Tipper, 1999). In this case, the way individuals allocate their attentional resources in response to a cuing stimulus is shaped by intrinsic structural features of the object rather than by the simple spatial information it conveys. According to neuroimaging evidence, space-based and object-centered attention mechanisms would be mainly served by common brain regions primarily located in the parietal cortex. In particular, these brain regions would include the left lateral inferior parietal cortex, the left prefrontal cortex, the left and right medial superior parietal cortex and also the cerebellar vermis (Fink et al., 1997). In addition, other brain regions would be differently recruited for the two frames of reference. Indeed, while object-centered attention would also recruit the left striate and prestriate cortex, space-based attention would recruit regions located in the right hemisphere such as the inferior temporal/fusiform gyrus and the dorsolateral prefrontal cortex (Fink et al., 1997).

In the same vein, studies addressing the relationship between gaze cuing and frames of reference provided evidence that attentional shifts occur even when the head is not presented in the standard upright position (Bayliss et al., 2004; see also Bayliss and Tipper, 2006). In more detail, Bayliss et al. (2004) employed a standard gaze-cuing task in which a central face with direct gaze suddenly looked rightwards or leftwards. After that, a to-be-detected target was presented either in the right or in the left hemifield. The peculiarity of this task was that the facial stimulus could appear either in the canonical upright orientation, resulting in a face looking rightwards or leftwards, or rotated 90° clockwise or anti-clockwise, resulting in a face looking upwards or downwards. When the face was presented upright, participants were faster in detecting targets that appeared in the same spatial location indicated by eye gaze (space-based orienting). Intriguingly, when the face was presented rotated, participants were still faster in detecting targets that appeared in the spatial location that would have been looked by the face, had this been presented upright (object-centered orienting). For instance, faces rotated 90° clockwise with eye gaze directed downwards elicited faster responses for targets that appeared on the right than on the left part of the screen. On the contrary, faces rotated 90° anti-clockwise with eye gaze directed downwards elicited faster responses for targets that appeared on the left than on the right side of the screen. This pattern of results is in line with previous evidence that suggested that eye gaze direction and head orientation are computed in parallel (e.g., Langton et al., 2000) rather than sequentially (i.e., eye gaze direction first, followed by head orientation), as originally proposed by the pioneering studies conducted by Perrett et al. (1992). This would explain the presence of the gaze cuing effect even within the object-centered frame: in this case, individuals would tend to compute gaze direction as if the head was oriented upright, which is undoubtedly more likely to occur during everyday social interactions (see Bayliss and Tipper, 2006). From a neuroanatomical perspective, the computation of eye gaze and head directions would be mainly supported by the right superior temporal sulcus, a brain area heavily involved in face processing (e.g., Haxby et al., 2000). However, more work is needed in order to get a broader picture concerning the neural mechanisms underlying this social form of spatial orienting.

Interestingly, both space-based and object-centered attention components seem to be preserved in right hemisphere-damaged patients (e.g., Driver and Halligan, 1991; Behrmann and Tipper, 1999). For instance, Behrmann and Tipper (1999) presented right hemisphere-damaged patients with two disks connected by a line and placed one in the left hemifield and one in the right hemifield, and two squares placed one in the left hemifield and one in the right hemifield. In this frame, slower RTs were reported in response to targets that appeared on stimuli (i.e., both circles and squares) on the left rather that on the right. However, when the two disks inverted their spatial position by rotating of 180°, slower RTs were reported in response to targets that appeared on the right disk as compared to RTs in response to targets on the left disk. As for squares, which contrary to disks remained stationary, slower RTs continued to be reported in response to left targets. These intriguing results seem to confirm that right hemisphere-damaged patients can allocate visual attention in different frames simultaneously. However, to the best of our knowledge, so far no studies have investigated this ability in gaze cuing of attention.

The aim of the present study was, therefore, twofold. Firstly, we aimed to provide further evidence concerning gaze cuing of attention in right hemisphere-damaged patients. Contrary to previous studies using schematic faces as cuing stimuli (e.g., Vuilleumier, 2002; Bonato et al., 2009), here we employed 3D avatars with a greater degree of ecological validity that should make eye gaze stimuli particularly relevant. Indeed, according to recent evidence, the sensitivity to eye gaze direction seems to be decreased when line-drawn face stimuli – such as those employed both in Bonato’s and in Vuilleumier’s studies – are employed (Rossi et al., 2015). On the contrary, the use of 3D avatars should facilitate the emergence of a robust gaze cuing of attention maintaining, at the same time, a strict control on the physical features of the facial stimuli.

Secondly, we aimed to explore whether right hemisphere-damaged patients exhibit a specific difficulty in object-centered gaze cuing of attention which could not be detected in previous studies that invariably used upright faces (e.g., Vuilleumier, 2002). To this purpose, we exploited the paradigm devised by Bayliss et al. (2004). Because for clinical testing a slightly different experimental setting was employed, we first attempted to replicate the main findings observed by Bayliss et al. (2004) in a sample composed of young healthy individuals (Experiment 1). In more detail, we expected, in line with Bayliss et al. (2004), a reliable and comparable gaze cuing of attention irrespectively of whether facial stimuli were presented upright or rotated. The same task employed in Experiment 1 was then administered in Experiment 2 to a group of right hemisphere-damaged patients, and to a matched group of healthy controls. We focused on a sample of right hemisphere-damaged patients in keeping with early neuropsychological studies using spatial cuing procedures (e.g., Posner et al., 1984). In addition, we included patients displaying diffused lesions and did not address specific brain areas because neuroimaging evidence suggests that a wide neural circuitry is involved in face processing and social attention (e.g., Allison et al., 2000; Haxby et al., 2000; Callejas et al., 2014). If right hemisphere-damaged patients process eye gaze stimuli within different frames of reference, then gaze cuing of attention should emerge irrespectively of head orientation. On the contrary, if right hemisphere-damaged patients process eye gaze stimuli only within a canonical framing in which head stimuli are presented upright, then gaze cuing of attention should be expected only within this frame. In both cases, these results coming from neuropsychological patients could hopefully provide new insights concerning both the behavioral mechanisms and the neural underpinnings of the space-based and the object-centered gaze cuing of attention.

## Experiment 1: Young Healthy Adults

### Materials and Methods

#### Participants

Twenty-six first-year undergraduate students (*Mean age* = 19.27 years, SD = 0.604, 5 males, 4 left handed) enrolled at the University of Padova participated in the experiment as part of course requirements. All participants were naïve to the purpose of the experiment and provided a written consent. The study was approved by the Ethics Committee for Psychological Research at the University of Padova and it was conducted in accordance with the Declaration of Helsinki.

#### Stimuli and Apparatus

Face stimuli consisted of eight 3D full-color avatars (4 males and 4 females) created through FaceGen 3.1. For each face there were three versions: one with direct gaze, one with gaze averted rightwards and one with gaze averted leftward. Faces lacked distracting elements such as hair and clothes (see also Pavan et al., 2011).

Stimulus presentation and data collection were handled through a laptop PC running E-prime 1.1. Participants sat 57 cm from the monitor (1024 × 768 pixels, 60 Hz) on which stimuli were presented against a gray background (*R* = 180, *G* = 180, *B* = 180).

#### Procedure

**FIGURE 1:**
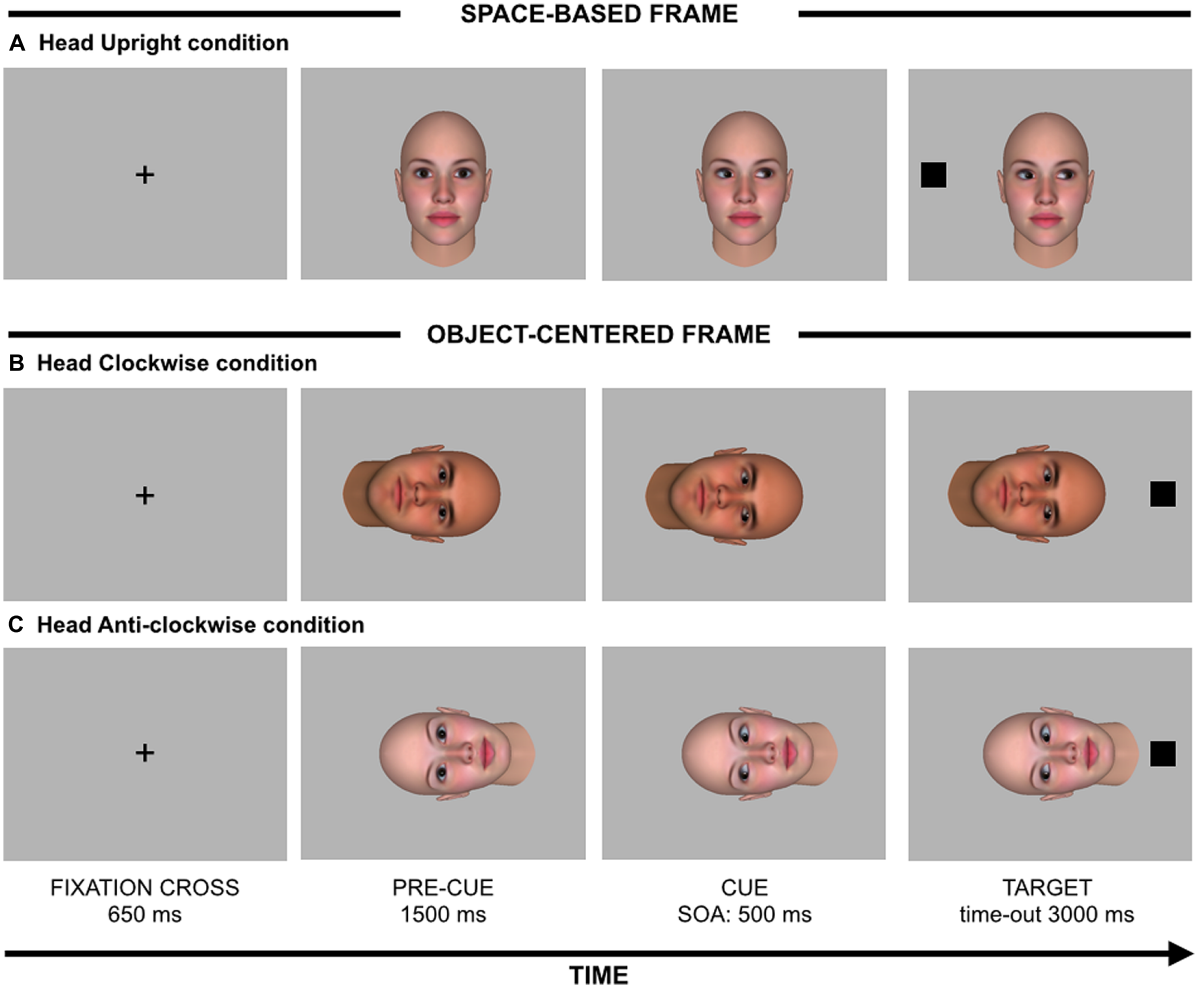
**Stimuli (not drawn to scale) and sequence of events for **(A)** an incongruent trial with a head oriented upright (space-based frame), **(B)** a congruent trial with a head oriented clockwise (object-centered frame), and **(C)** an incongruent trial with a head oriented anti-clockwise (object-centered frame)**.

The procedure was similar to that used by Bayliss et al. (2004). Each trial began with a centrally placed black fixation cross (1°height × 1°width) for 650 ms (see **Figure 1**), followed by a face with direct gaze which served as a pre-cue. Depending on condition, this face could appear oriented in three different orientations: upright (space-based frame; 16.8°height × 14.4°width), rotated 90° clockwise or anti-clockwise (object-centered frame). In these two latter orientations, the rotation was centered on the middle of the eyes. After 1500 ms, the same face was presented with gaze averted either rightwards or leftward, which served as a spatial cue. After a fixed 500-ms stimulus onset asynchrony (SOA), a black square (1.3°height × 1.3°width) which served as target appeared 13.3° to the right or to the left with respect to the center of the screen. Participants were instructed to detect the target by pressing the space bar as fast as possible with the index finger of their dominant hand. In the space-based frame, a congruent trial occurred when the target appeared on the same spatial location gazed at by the upright face stimulus. In the object-centered frame, a congruent trial occurred when the target appeared on the same spatial location looked at by the rotated face stimulus had this been presented upright (see **Figure 1**). Both cue and target stimuli remained visible until the participant’s response or until 3000 ms elapsed, whichever came first. We also included catch trials to prevent anticipatory responses. In the case of a catch trial, the target did not appear and participants were instructed to refrain from responding. The red words “NO RESPONSE” and “ERROR” were presented when participants did not respond within 3000 ms (i.e., missed responses) and when they responded on catch trials (i.e., false alarms), respectively.

On each trial, face frame (upright vs. rotated), gaze direction (left vs. right), and target location (left vs. right) were selected randomly. Each combination of these factors was presented an equal number of times. When the face was not upright, head was equally likely to be rotated clockwise or anti-clockwise. The participants were informed that head orientation and gaze direction were both uninformative about the spatial location of the upcoming target, which could appear either on the right or the left with the same probability. Moreover, they were also asked to maintain their eyes on the center of the screen for the whole duration of the experiment. There was a practice block composed of 9 target-present trials and 3 catch trials, followed by three experimental blocks each composed of 64 target-present trials and 16 catch trials. The whole experiment was composed of 240 experimental trials.

### Results

#### Data Reduction

Missed responses (0.24 % of trials) and false alarms (0.4 % of trials) were removed and, because of their low rate of frequency, they were not analyzed further. Anticipations, defined as RTs less than 100 ms and outliers, defined as RTs that fall 3 SD above the mean of each participant were also removed (1.5% of trials; see also Bonato et al., 2009).

#### Reaction Time Analysis

Reaction times for correct responses were analyzed using JASP 0.7 software (Love et al., 2015) through a repeated-measures ANOVA with cue-target spatial congruency (2: congruent vs. incongruent) and frame (2: spatial vs. object) as within-participant factors. Furthermore, in order to assess which model (i.e., H0 vs. H1) was more likely supported by the current data, the Bayes Factor (BF; e.g., Rouder et al., 2009) was also computed.

**FIGURE 2:**
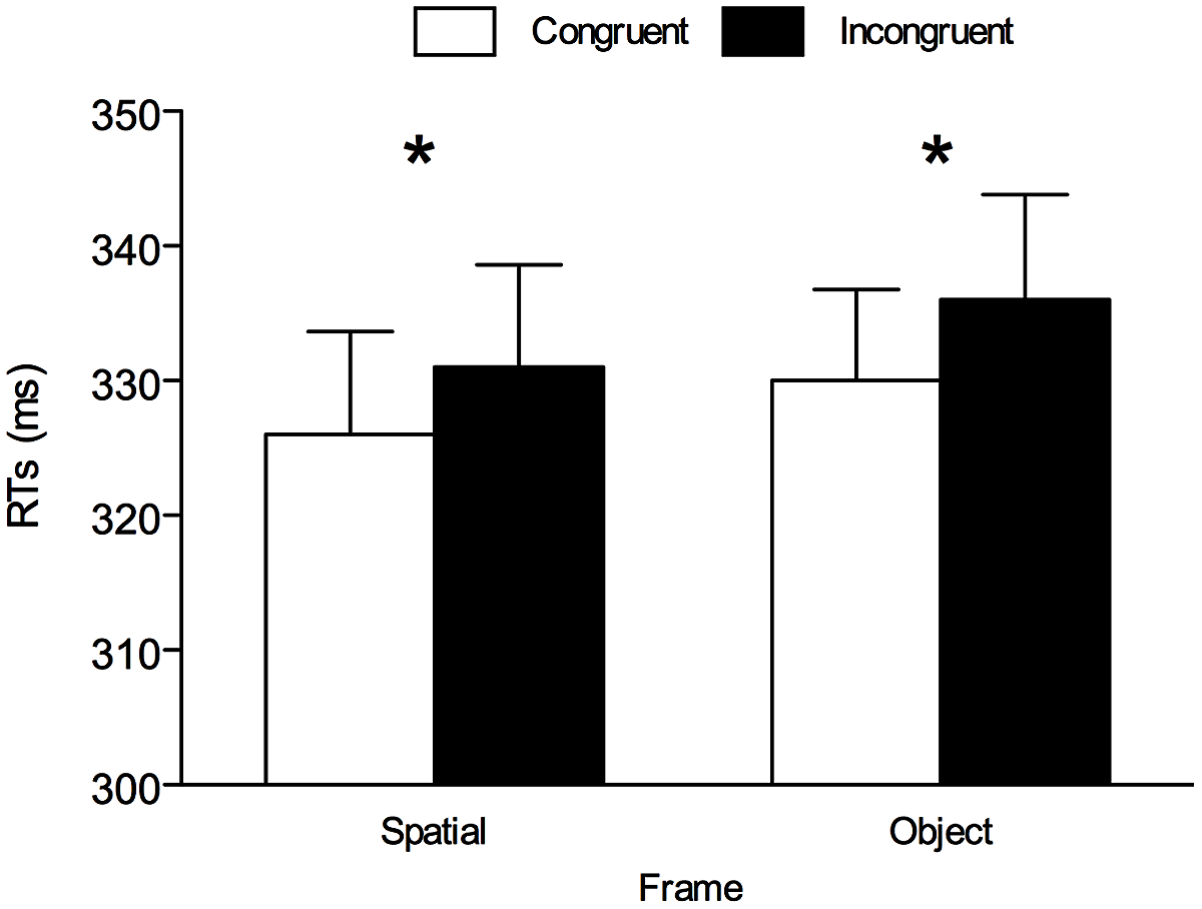
**Mean reaction times (RTs) for all conditions in Experiment 1.** Asterisks denote *p* < 0.05. Error bars are SEM.

The only significant main effect was cue-target spatial congruency, *F*(1,25) = 8.484, *p* = 0.007, $\eta_{p}^{2}$ = 0.253, confirming the presence of an overall gaze-cuing effect with shorter RTs on spatially congruent trials (*M* = 328 ms, SE = 7.06) than on spatially incongruent trials (*M* = 334 ms, SE = 7.64). The main effect of frame only approached significance, *F*(1,16) = 3.966, *p* = 0.057, $\eta_{p}^{2}$ = 0.137 (see **Figure 2**). Importantly, the cue-target spatial congruency × frame interaction was not significant (*F* < 1, *p* = 0.894), suggesting a comparable gaze-cuing effect in each frame. In line with this, BF analysis showed that the model with only main effects, BF_10_ = 9.829, was preferable over the model also including the interaction, BF_10_ = 2.632. For completeness, one-tailed paired *t*-tests were performed between congruent and incongruent trials divided by frame. These analyses revealed a significant gaze-cuing effect both in the space-based frame, *t*(25) = 2.147, *p* = 0.021, *d_z_* = 0.421, and in the object-centered frame, *t*(25) = 2.097, *p* = 0.023, *d_z_* = 0.411 (see **Figure 2**). BF analysis showed that both in the space-based frame, BF_10_ = 2.610, and in the object-centered frame, BF_10_ = 2.843, the model supporting H1 (i.e., the presence of the gaze cuing effect) was preferable over the model supporting H0 (i.e., the absence of the gaze cuing effect).

Overall, this pattern of results is fully consistent with the findings reported by Bayliss et al. (2004), and it confirms that the paradigm used here is suitable for revealing both a space-based and an object-centered component in gaze cuing. Therefore, the same paradigm was also used in Experiment 2 in order to assess whether those two components emerged in right hemisphere-damaged patients.

## Experiment 2: Right Hemisphere-Damaged Patients vs. Healthy Matched Controls

### Materials and Methods

#### Participants

**FIGURE 3:**
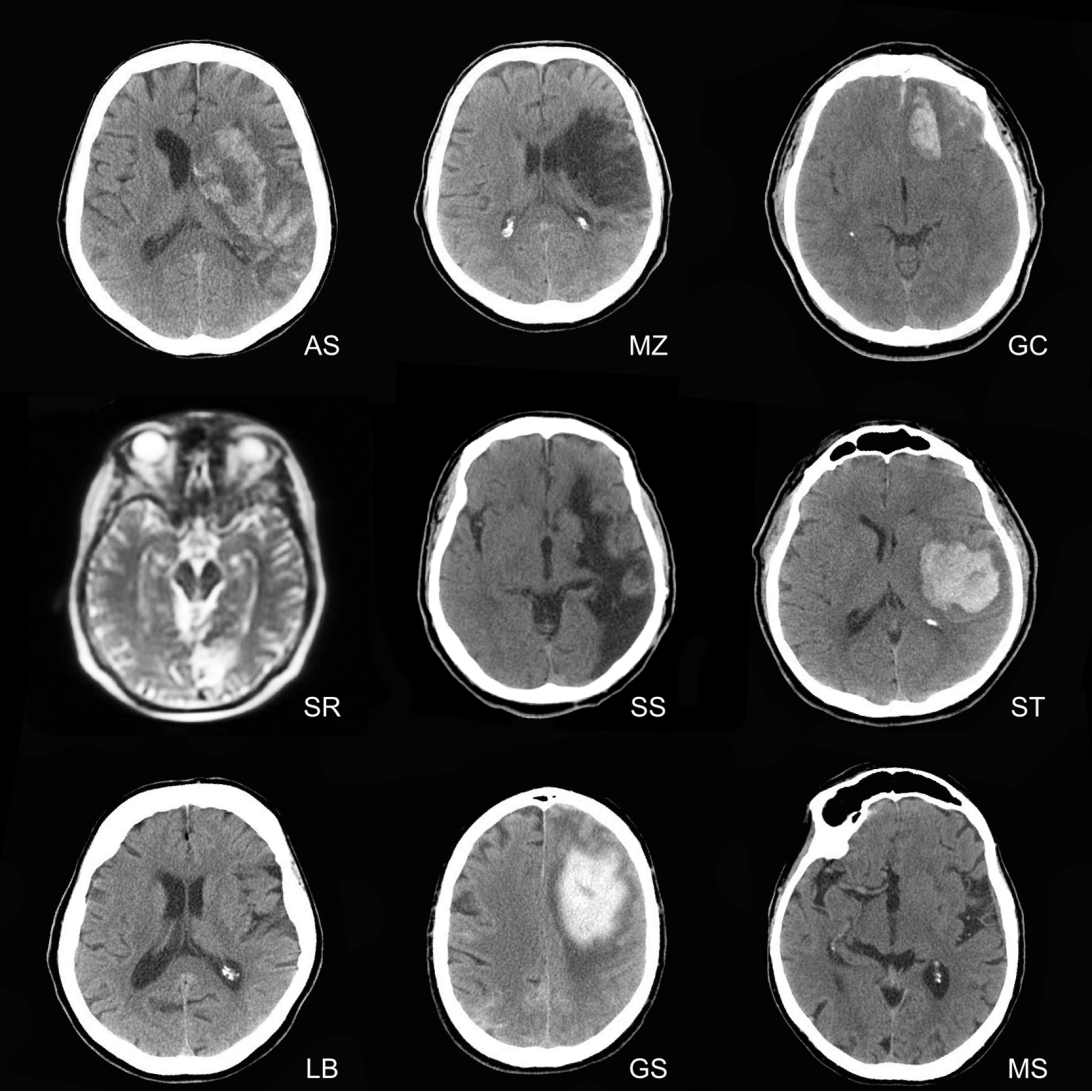
**Magnetic resonance imaging and Computed Tomography scans of right hemisphere-damaged patients.** The right hemisphere is displayed on the right in each brain scan.

The experimental group was composed of eleven individuals recruited in a public clinic located in northern Italy. They were recruited on the basis of the lack of mental retardation and a diagnosis of brain lesions limited to the right hemisphere, in accordance with board-certified neuroradiological reports (see **Figure 3**). Two patients were excluded from the analyses, because of difficulties in understanding the instructions and completing the experiment. The final sample was thus composed of nine patients (*Mean age* = 63 years, SD = 15.2, *mean education* = 7.56 years, SD = 2.65, three females, all right handed). Demographic and clinical information of patients is reported in **Table 1**.

**Table 1:** Demographic and clinical data for right hemisphere-damaged patients in Experiment 2.

Patient	AS	MZ	GC	SR	SS	ST	LB	GS	MS
Age (years)	76	64	50	48	39	60	81	67	82
Education (years)	5	10	7	13	7	8	5	8	5
Gender	Female	Male	Male	Male	Male	Male	Female	Male	Female
Handedness	R	R	R	R	R	R	R	R	R
Lesion site^a^	P, T	F, P, T	F	O	F, P, T	IP	TN	PA, F	P, O, CN
Etiology^b^	I	I	H	I	H	H	I	H	I
Hospitalization	Yes	No	No	Yes	No	Yes	Yes	Yes	No
Time since lesion^c^ (days)	79	835	131	64	447	60	23	62	7

*^a^Confirmed by neuroradiological reports. CN, caudate nucleus; F, frontal; IP, intraparenchymal; O, occipital; P, parietal; PA, parenchymal; T, temporal; TN, thalamic nucleus.*

*^b^H, hemorrhagic; I, ischemic. ^c^Acute event.*

The control group was composed of 9 healthy individuals (*Mean age* = 63.11 years, SD = 15.44, *mean education* = 11.22 years, SD = 5.26, three females, all right handed), recruited in the local population to match the patients for age, education, gender, and handedness. Two-tailed paired *t*-tests between mean age, *t*(16) = 0.15, *p* = 0.988, and education, *t*(16) = 1.867, *p* = 0.087, of patients and controls confirmed that the two groups were roughly comparable. An interview was administered to all of them in order to exclude previous history of neurological disease.

#### Stimuli and Apparatus

Stimuli and apparatus were identical to those employed in Experiment 1.

#### Procedure

The procedure was identical to that employed in Experiment 1.

### Results

#### Data Reduction

Data reduction was the same as that adopted in Experiment 1. Missed responses (6.35% of trials) and false alarms (2.08% of trials) were removed and analyzed separately. Anticipations, defined as RTs less than 100 ms and outliers, defined as RTs that fall 3 SD above the mean of each participant were also removed (1.24% of trials).

#### Reaction Time Analysis

Reaction times for correct responses were analyzed using JASP 0.7 software (Love et al., 2015) through a mixed-design ANOVA with cue-target spatial congruency (2: congruent vs. incongruent) and frame (2: spatial vs. object) as within-participant factors. Hemifield (2: right vs. left) was also included as within-participant factor in order to investigate the potential presence of lateralized effects in right hemisphere-damaged patients (see also Bonato et al., 2009). Group (2: right hemisphere-damaged patients vs. healthy controls) was included as between-participant factor.

The main effect of cue-target spatial congruency was significant, *F*(1,16) = 5.568, *p* = 0.031, $\eta_{p}^{2}$ = 0.258, confirming the presence of an overall gaze-cuing effect with shorter RTs on spatially congruent trials (*M* = 697 ms, SE = 68.24) than on spatially incongruent trials (*M* = 725 ms, SE = 70.96), as well as the main effect of hemifield, *F*(1,16) = 8.738, *p* = 0.009, $\eta_{p}^{2}$ = 0.353, owing to shorter RTs when the target appeared on the right hemifield (*M* = 634 ms, SE = 57.12) rather than on the left hemifield (*M* = 788 ms, SE = 87.91). The main effect of group was also significant, *F*(1,16) = 6.116, *p* = 0.025, $\eta_{p}^{2}$ = 0.277, owing to shorter RTs in healthy participants (*M* = 539 ms, SE = 63.22) than in right hemisphere-damaged patients (*M* = 883 ms, SE = 123.53). The cue-target spatial congruency × hemifield interaction was significant, *F*(1,16) = 9.998, *p* = 0.006, $\eta_{p}^{2}$ = 0.385, as well as the hemifield × group interaction, *F*(1,16) = 10.244, *p* = 0.006, $\eta_{p}^{2}$ = 0.390, while the cue-target spatial congruency × group interaction was not significant, *F*(1,16) = 3.269, *p* = 0.089, $\eta_{p}^{2}$ = 0.190.

**FIGURE 4:**
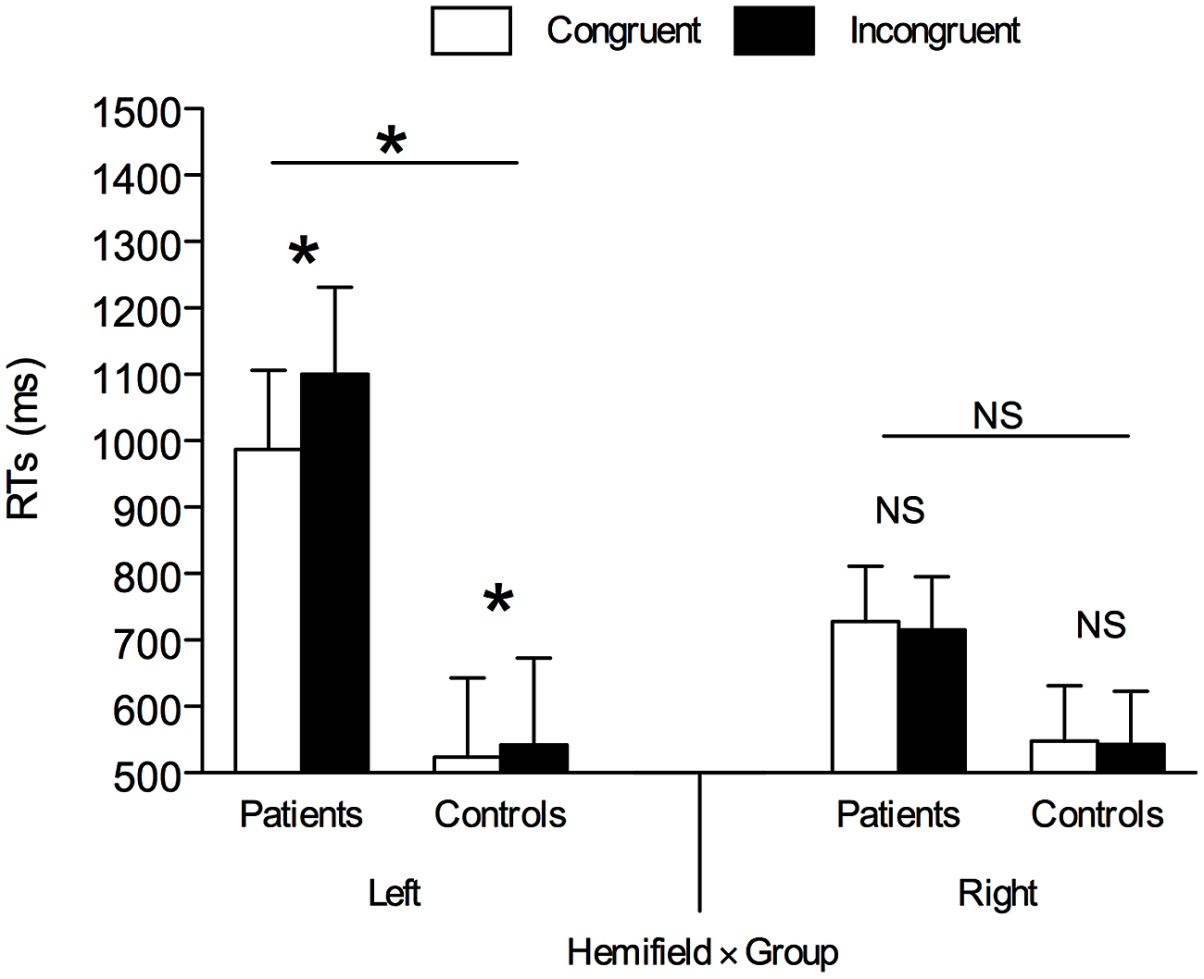
**Mean RTs in left and right hemifield in right hemisphere-damaged patients vs. healthy controls.** Asterisks denote *p* < 0.05. NS, Non-Significant; Error bars are SEM.

Importantly, all the previous two-way interactions were qualified by the cue-target spatial congruency × hemifield × group three-way interaction, *F*(1,16) = 4.713, *p* = 0.045, $\eta_{p}^{2}$ = 0.228. This three-way interaction was further analyzed through two separate ANOVAs as a function of the hemifield with cue-target spatial congruency as within-participant factor and group as between-participant factor. As for targets appearing on the right hemifield, the main effect of group was not significant, *F*(1,16) = 2.374, *p* = 0.143, $\eta_{p}^{2}$ = 0.129, but the means indicated that RTs were shorter in healthy participants (*M* = 546 ms, SE = 65.19) than in right hemisphere-damaged patients (*M* = 722 ms, SE = 93.82). All other results were non-significant (*F*s < 1, *p*s > 0.436; BF_10_s < 1). Nevertheless, for completeness, one-tailed paired *t*-tests between congruent and incongruent trials divided by group confirmed that the gaze-cuing effect was absent both in healthy controls, *t*(8) = –0.605, *p* = 0.281, *d_z_* = –0.202, and in right hemisphere-damaged patients, *t*(8) = –0.620, *p* = 0.276, *d_z_* = –0.207 (see **Figure 4**). BF analysis showed that both in healthy controls, BF_10_ = 0.222, and in right hemisphere-damaged patients, BF_10_ = 0.221, the model supporting H0 was preferable over the model supporting H1.

As for targets appearing on the left hemifield, the main effect of cue-target spatial congruency was significant, *F*(1,16) = 9.951, *p* = 0.006, $\eta_{p}^{2}$ = 0.383, owing to shorter RTs on spatially congruent trials (*M* = 756 ms, SE = 83.98) than on spatially incongruent trials (*M* = 821 ms, SE = 92.84), as well as the main effect of group, *F*(1,16) = 8.425, *p* = 0.010, $\eta_{p}^{2}$ = 0.345, owing to overall shorter RTs in healthy controls (*M* = 533 ms, SE = 61.61) than in right hemisphere-damaged patients (*M* = 1043 ms, SE = 164.68). The cue-target spatial congruency × group interaction was also significant, *F*(1,16) = 5.170, *p* = 0.037, $\eta_{p}^{2}$ = 0.244. One-tailed paired *t*-tests between congruent and incongruent trials divided by group indicated that the gaze-cuing effect was present both in healthy controls, *t*(8) = 2.244, *p* = 0.028, *d_z_* = 0.748, and in patients, *t*(8) = 2.768, *p* = 0.012, *d_z_* = 0.923, but the effect was much stronger in the latter case (18 ms vs. 112 ms)^1^. BF analysis showed that both in healthy controls, BF_10_ = 3.261, and in right hemisphere-damaged patients, BF_10_ = 6.195, the model supporting H1 was preferable over the model supporting H0.

Because Bonato et al. (2009) documented the presence of a disengagement deficit in their patients, at least when arrow cues were used, we also implemented their formula to explore whether such phenomenon was also evident in our sample. To this end, the gaze cuing effect (RT_incongruent_ – RT_congruent_) was separately calculated for targets appearing in the left and in the right hemifield, and the difference between them was finally computed (Cuing_left_ – Cuing_right_). A one-sample *t*-test showed that this index was significantly different from zero, *t*(8) = 2.761, *p* = 0.025, *d_z_* = 0.920, thus confirming the presence of a disengagement deficit in our sample of right hemisphere-damaged patients. BF analysis showed that the model supporting H1 was preferable over the model supporting H0, BF_10_ = 3.134.

Importantly, all the interactions involving cue-target spatial congruency and frame were not significant (*F*s < 1, *p*s > 0.443, BF_10_s < 1), suggesting a comparable gaze-cuing effect for the two frames (see **Table 2**). Nevertheless, for completeness, one-tailed paired *t*-tests were performed between congruent and incongruent trials divided by frame and group. These analyses were carried out only for target appearing on the left hemifield since the gaze-cuing effect was observed only there. As for healthy controls, a significant gaze cuing emerged in the space-based frame, *t*(8) = 2.325, *p* = 0.024, *d_z_* = 0.775, while in the object-centered frame the effect was not significant, *t*(8) = 0.719, *p* = 0.246, *d_z_* = 0.240, although means were in the expected direction, with shorter RTs on congruent trials (*M* = 534 ms, SE = 59.17) than on incongruent trials (*M* = 540 ms, SE = 63.41). BF analysis showed that the model supporting H1 was preferable over the model supporting H0 in the space-based frame, BF*_10_* = 3.601, while in the object-centered frame this was less evident, BF*_10_* = 0.587. Patients oriented attention in response to gaze both in the space-based frame, *t*(8) = 1.866, *p* = 0.049, *d_z_* = 0.622, and in the object-centered frame, *t*(8) = 2.258, *p* = 0.027, *d_z_* = 0.753. BF analysis showed that the model supporting H1 was preferable over the model supporting H0 both in the space-based frame, BF*_10_* = 2.066, and in the object-centered frame, BF*_10_* = 3.320.

**Table 2:** Mean reaction times (RTs; ms) and percentage of errors (%E) for all conditions in Experiment 2.

Group	Scores		Space-based frame				Object-centered frame			

			**Left hemifield**		**Right hemifield**		**Left hemifield**		**Right hemifield**	
				
			**C**	**I**	**C**	**I**	**C**	**I**	**C**	**I**
Healthy individuals (control group)	RTs		514 (58)	544 (67)	531 (69)	537 (68)	534 (59)	540 (63)	566 (65)	549 (60)
	%E	MR	0 (0)	0.46 (0.46)	0.46 (0.46)	0 (0)	0 (0)	0 (0)	0 (0)	0 (0)
		FA	1.39 (1.39)				0.46 (0.46)			
Right hemisphere-damaged patients	RTs		983 (153)	1113 (176)	702 (93)	692 (82)	991 (171)	1087 (173)	755 (101)	738 (108)
	%E	MR	16 (6.66)	20 (6.03)	7 (3.43)	6 (4.57)	20 (8.1)	18 (5.5)	8 (4.89)	6 (3.26)
		FA	4.17 (3.68)				2.32 (1)			

*Values in brackets are SEM. C, congruent trial; I, incongruent trial; MR, Missed Responses; FA, False Alarms.*

#### Error Analysis

Missed responses were analyzed through a mixed-effect logit model (e.g., Jaeger, 2008). In this analysis, cue-target spatial congruency, frame, hemifield, and group were treated as fixed effects, and participant was treated as random effect. In a first model, both main effects and interactions were tested. Under these circumstances, no significant results emerged (*p*s > 0.152). For this reason, a second model was implemented considering only the main effects. In this case, the main effect of hemifield was significant, *b* = –1.440, SE = 0.173, *z* = –8.316, *p* < 0.001, owing to more missed responses when the target appeared on the left hemifield than on the right hemifield. The main effect of group was also significant, *b* = –5.094, SE = 1.236, *z* = –4.121, *p* < 0.001, owing to more missed responses in right hemisphere-damaged patients than in healthy controls (see **Table 2**). Other main effects were not significant (*p*s > 0.635). Model comparison was performed following the guidelines proposed by Bolker et al. (2009). BIC values suggested that evidence supporting the model with only main effects (BIC = 1132.8) over the model in which also interactions were considered (BIC = 1213.0) was very strong (ΔBIC = 81; see Raftery, 1995). In the same fashion, the likelihood ratio test indicated that the model in which also interactions were considered did not provide additional information with respect to the model with only main effects, χ^2^ (11) = 9.691, *p* = 0.558.

Similarly, we also analyzed false alarms in catch trials through a mixed-effect logit model with frame and group as fixed effects, and participant as random effect. In a first model, both main effects and interactions were tested. Under these circumstances, no significant results emerged (*p*s > 0.258). For this reason, a second model was implemented considering only the main effects. In this case, the main effect of group was significant, *b* = –4.992, SE = 1.209, *z* = –4.130, *p* < 0.001, owing to more false alarms in right hemisphere-damaged patients than in healthy controls (see **Table 2**). The main effect of frame was not significant (*p* = 0.647). Model comparison was performed following the guidelines proposed by Bolker et al. (2009). BIC values suggested that evidence supporting the model with only main effects (BIC = 1195.2) over the model in which also interactions were considered (BIC = 1200.4) was positive (ΔBIC = 5; see Raftery, 1995). In the same fashion, the likelihood ratio test indicated that the model in which also interactions were considered did not provide additional information with respect to the model with only main effects, χ^2^(1) = 2.946, *p* = 0.09.

## Discussion

The ability to orient attention in response to spatial signals provided by our conspecifics represents a key element of human behavior (e.g., Baron-Cohen, 1995), and research has focused on both the cognitive aspects of the phenomenon as well as on the neural underpinnings that would serve gaze cuing of attention. Neuroimaging studies (e.g., Hietanen et al., 2006; Tipper et al., 2008; Callejas et al., 2014) indicate that this form of social orienting involves brain areas mainly localized in the right hemisphere. The possible existence of a broad neural network devoted to gaze cuing of attention emerged also from neuropsychological studies (e.g., Vuilleumier, 2002; Bonato et al., 2009) in which right hemisphere-damaged patients often showed a relatively spared ability to shift attention toward spatial locations indicated by eye gaze stimuli, at least when lesions do not specifically involve the superior temporal gyrus and the superior temporal sulcus (Akiyama et al., 2006).

The general aim of the present study was to provide further evidence on gaze cuing of attention in a sample of right hemisphere-damaged patients. Unlike previous neuropsychological studies, which only focused on the space-based component of gaze cuing of attention, here we also explored the object-centered component of this form of orienting. To reach this goal, in two experiments, we employed a task similar to that devised by Bayliss et al. (2004) in which a centrally placed head with averted gaze, displayed upright (space-based orienting) or rotated 90° clockwise or anti-clockwise (object-centered orienting), preceded the onset of a target that could appear either in the right or in the left hemifield. In Experiment 1, we tested a sample of young healthy individuals. The same task was also administered in Experiment 2 to a sample of right hemisphere-damaged patients compared with a matched group of healthy individuals.

As for the overall gaze-cuing effect, the results stemming from right hemisphere-damaged patients were, on the whole, consistent with those reported by Vuilleumier (2002) and Bonato et al. (2009) in that the ability to shift attention in response to eye gaze stimuli was preserved. However, gaze cuing was significant only when targets appeared in the left hemifield (see Bonato et al., 2009). This finding is in line with previous evidence according to which right hemisphere-damaged patients often suffer from a disengagement deficit of attention following a spatially incongruent cue pointing to the right visual hemifield (e.g., Posner et al., 1984; Bartolomeo et al., 2001; for a review, see Bartolomeo and Chokron, 2002). In other words, responses would be particularly slowed down when targets are presented in the contralesional side (i.e., left visual hemifield) after a spatial cue that pushed attention towards the ipsilesional side (i.e., right visual hemifield). The presence of a disengagement deficit seems more frequent in response to peripheral cues (see Losier and Klein, 2001), although it has also been documented in response to centrally placed arrow cues (Bonato et al., 2009; Olk et al., 2010) but not in response to centrally placed eye gaze cues (Bonato et al., 2009). Strikingly, our results provide first evidence of a disengagement deficit in response to centrally placed gaze cues in patients with a damage to the right hemisphere. Despite the comparison between our results and those reported by Bonato et al. (2009) must be taken with caution – due to relevant differences in both the methodology and the clinical samples – the discrepant pattern may be tentatively explained by taking into account the specific type of eye gaze stimuli used in the two studies. Indeed, while in the present study we employed 3D avatars that suddenly moved their eyes rightwards or leftwards – mimicking actual social interactions – in Bonato et al. (2009), participants were presented with schematic eyes in isolation (i.e., not embedded within a face) with static pupils oriented rightwards or leftward. Interestingly, in the present study, also participants from the control group showed a reliable gaze cuing of attention only for targets appearing in the left hemifield, even though this effect was significantly larger among right hemisphere-damaged patients (i.e., 112 ms) as compared to healthy participants (i.e., 18 ms, a magnitude which is in line with previous reports; e.g., Friesen and Kingstone, 1998). Gaze cuing of attention only in response to targets presented in the left hemifield has also been documented in a recent study, conducted by Marotta et al. (2012a), that administered to healthy participants a similar paradigm to that employed here. In more detail, Marotta et al. (2012a) asked participants to detect a target, which could appear rightwards or leftwards, in the presence of centrally presented task-irrelevant arrow and eye gaze cues oriented rightwards or leftwards. Strikingly, while a reliable arrow cuing of attention emerged irrespectively of whether the target appeared in the left or in the right hemifield, a reliable gaze cuing emerged only in response to targets appearing in the left hemifield. The authors interpreted this pattern of results as likely reflecting the specialization of the right hemisphere in face processing (e.g., Ojemann et al., 1992). This conclusion is also consistent with a previous study conducted in healthy individuals that suggests that while symbolic spatial cuing of attention – such as the one obtained with arrows – would be supported by brain mechanisms spread bilaterally, gaze cuing of attention would be specifically supported by brain areas located in the right hemisphere (Greene and Zaidel, 2011). Moreover, as discussed in the introduction, this scenario is also supported by evidence coming from split-brain patients who exhibited arrow cuing of attention in response to targets presented bilaterally in the face of a gaze cuing of attention limited to targets presented to the left (Kingstone et al., 2000; Ristic et al., 2002). However, the scarcity of evidence on this topic invites to take this conclusion with caution and future studies are necessary in order to test exhaustively the possible different contribution that the two hemispheres provide to the social and the symbolic cuing of attention.

One of the major goals of the present study, was also to address the potential role of the frame of reference (i.e., space-based vs. object-centered) in shaping attentional orienting. In Experiment 1, we replicated the pattern of results reported by Bayliss et al. (2004) in a sample of young healthy individuals. Indeed, a reliable and comparable gaze cuing of attention emerged irrespectively of head orientation. Importantly, in Experiment 2, a similar pattern emerged, namely a gaze cuing of attention of similar magnitude was observed both under space-based and object-centered frames, at least when targets were presented in the left hemifield. This finding provides further evidence supporting the notion that, also in right hemisphere-damaged patients, visual attention can operate within different frames of reference and suggests that this ability is not limited to symbolic cues (e.g., Driver and Halligan, 1991; Behrmann and Tipper, 1999) but it extends to a social stimulus such as eye gaze. An intriguing research question that could be addressed in future studies is to explore whether space-based orienting and object-centered orienting are sensitive to context information such as in the case in which reference objects (e.g., placeholders) are presented – or not – in the periphery. Indeed, recent evidence has reported that, when in a gaze cuing task no placeholders are presented, the gaze cuing effect emerges not only in response to a specific spatial location but instead it is also detectable in response to targets appearing in different spatial locations within the cued hemifield. On the contrary, when placeholders are used, the gaze cuing effect emerges only in response to targets appearing inside the placeholder (Wiese et al., 2013; see also Marotta et al., 2012b for similar results). Following this rationale, it would be interesting to employ a modified version of the paradigm adopted in the present study in which the presence of peripheral placeholders is manipulated. Following the results reported by Wiese et al. (2013), in the presence of placeholders the gaze cuing effect should emerge only within the space-based frame of reference.

Future work could be carried out also to overcome some limitations that characterize the present study. First of all, at the time of testing we have been unable to administer standardized measures of neuropsychological tests to all the individuals of our clinical sample. This prevented us from assessing the potential presence of hemispatial neglect and its possible role in shaping socio-attentional mechanisms. For instance, a neuropsychological assessment tool such as the Behavioral Intentional Test (e.g., Wilson et al., 1987) could be employed in order to unveil any potential relationship between symptom variables and gaze cuing of attention within different frames of reference. Furthermore, to what concerns the methodological aspects of the paradigm employed here, it is important to highlight the fact that we used a fixed 500-ms SOA. The main reason for this choice was for coherence with the original study of Bayliss et al. (2004) in which the same SOA was used. However, future studies could employ a broader range of SOA in order to properly assess the temporal dynamics underlying the gaze cuing effect within different frame of reference. Bayliss and Tipper (2006), who employed a similar paradigm as that proposed in Bayliss et al. (2004), used two SOAs of about 200 and 500 ms. In both cases, they reported both space-based and object-centered gaze cuing of attention, but at the shorter SOA this effect was overall weaker, especially within the object-centered frame (gaze cuing effect = 4 ms) as compared to the spatial-based frame (gaze cuing effect = 9 ms).

In summary, our results confirm the presence of spared gaze cuing of attention in right hemisphere-damaged patients, and are overall consistent with previous studies (e.g., Vuilleumier, 2002; Bonato et al., 2009). Furthermore, they provide first evidence that gaze cuing of attention in right hemisphere-damaged patients can operate within different frames of reference. Previous studies only focused on symbolic spatial cues (e.g., Driver and Halligan, 1991; Behrmann and Tipper, 1999), and we here show that object-centered orienting is preserved also for a relevant social cue such as eye gaze. Because the study of the neural underpinnings underlying gaze cuing of attention is still an ongoing endeavor, further studies are necessary in order to achieve an exhaustive scenario concerning the brain areas involved in this form of social orienting. In this regard, the adoption of a causal approach based on neuropsychological evidence, aimed to address the effects of more focal lesions not limited to the right hemisphere (e.g., Vecera and Rizzo, 2004, 2006), represents a fruitful path for future research.

## Conflict of Interest Statement

The authors declare that the research was conducted in the absence of any commercial or financial relationships that could be construed as a potential conflict of interest.

## Footnotes

1 The fact that gaze cuing emerged only when target appeared in the left hemifield could be expected for right hemisphere-damaged patients. A similar pattern for healthy controls, however, should not come as a surprise, in that a recent study found that even in healthy individuals gaze cuing was detectable only when targets appeared on the left hemifield (Marotta et al., 2012a), likely reflecting the cerebral hemispheric specialization for face processing. In order to test whether the gaze-cuing effect emerged only in the left hemifield also in Experiment 1, we further analyzed RTs data from Experiment 1 through a repeated-measures ANOVA with cue-target spatial congruency (2: congruent vs. incongruent), frame (2: space-based vs. object-centered) and hemifield (2: right vs. left) as within-participant factors. The results remained virtually unchanged. Indeed, the only significant result was the cue-target spatial congruency main effect, *F*(1,25) = 8.534, *p* = 0.007, $\eta_{p}^{2}$ = 0.254. The cue-target spatial congruency × hemifield interaction did not reach significance, *F*(1,25) = 1.289, *p* = 0.267, $\eta_{p}^{2}$ = 0.049, although the gaze cuing effect (RT_incongruent_ – RT_congruent_) was larger for targets appearing on the left hemifield (7 ms) as compared to the right hemifield (3 ms). Interestingly, one-sample *t*-tests confirmed that the gaze-cuing effect was statistically different from zero in the left hemifield, *t*(25) = 2.870, *p* = 0.008, *d_z_* = 0.563, but not in the right hemifield, *t*(25) = 1.165, *p* = 0.255, *d_z_* = 0.228. In line with this, BF analysis showed that the model supporting H1 was preferable over the model supporting H0 in the left hemifield, BF_10_ = 5.566, but not in the right hemifield, BF_10_ = 0.381.
